# Parameter estimate of signal transduction pathways

**DOI:** 10.1186/1471-2202-7-S1-S6

**Published:** 2006-10-30

**Authors:** Ivan Arisi, Antonino Cattaneo, Vittorio Rosato

**Affiliations:** 1 European Brain Research Institute, Via Fosso del Fiorano 64, Roma, Italy; 2 Lay Line Genomics SpA, S.Raffaele Science Park, Castel Romano, Italy; 3 International School of Advanced Studies (SISSA/ISAS), Biophysics Dept., Via Beirut 2-4, Trieste, Italy; 4 ENEA, Casaccia Research Center, Computing and Modelling Unit, Via Anguillarese 301, S.Maria di Galeria, Italy; 5 Ylichron Srl, c/o ENEA, Casaccia Research Center, Via Anguillarese 301, S.Maria di Galeria, Italy

## Abstract

**Background:**

The "inverse" problem is related to the determination of unknown causes on the bases of the observation of their effects. This is the opposite of the corresponding "direct" problem, which relates to the prediction of the effects generated by a complete description of some agencies. The solution of an inverse problem entails the construction of a mathematical model and takes the moves from a number of experimental data. In this respect, inverse problems are often ill-conditioned as the amount of experimental conditions available are often insufficient to unambiguously solve the mathematical model. Several approaches to solving inverse problems are possible, both computational and experimental, some of which are mentioned in this article. In this work, we will describe in details the attempt to solve an inverse problem which arose in the study of an intracellular signaling pathway.

**Results:**

Using the Genetic Algorithm to find the sub-optimal solution to the optimization problem, we have estimated a set of unknown parameters describing a kinetic model of a signaling pathway in the neuronal cell. The model is composed of mass action ordinary differential equations, where the kinetic parameters describe protein-protein interactions, protein synthesis and degradation. The algorithm has been implemented on a parallel platform. Several potential solutions of the problem have been computed, each solution being a set of model parameters. A sub-set of parameters has been selected on the basis on their small coefficient of variation across the ensemble of solutions.

**Conclusion:**

Despite the lack of sufficiently reliable and homogeneous experimental data, the genetic algorithm approach has allowed to estimate the approximate value of a number of model parameters in a kinetic model of a signaling pathway: these parameters have been assessed to be relevant for the reproduction of the available experimental data.

## Background

The "inverse" problem is related to the determination of unknown causes on the bases of the observation of their effects. This is the opposite of the corresponding "direct" problem, which relates to the prediction of the effects generated by a complete description of some agencies. Typical inverse problems in electrocardiology are related to the modelling of the human heart functional structure from surface electrocardiogram signals (ECG) [1]; similar situations are encountered in magnetoencephalography (MEG) and electroencephalography (EEG) [2,3]. In biology, a classical example of the "inverse" approach is the reconstruction of the three-dimensional structure of macromolecules, using either x-ray diffraction, nuclear magnetic resonance (NMR) or prediction models [4-6]. Another typical biological application of inverse approaches is the reconstruction of gene-regulatory networks [7,8].

The solution of an inverse problem entails the construction of a mathematical model and takes the moves from a number of experimental data. In this respect, inverse problems are often ill-conditioned as the amount of experimental conditions available are often insufficient to unambiguously solve the mathematical model. Moreover, as model construction usually depends upon the minimization of specific functions, such as the system energy or the difference between the model prediction and some given experimental results, its solution does not necessarily lead to a single global optimal solution but to a set of optimal solutions, defining what is called the "Pareto optimal frontier" in the space of solutions [9]. Additional experimental constraints or theoretical methods are thus necessary to further select within the solutions set. Typical inverse problems concerns essentially the detailed determination of biochemical mechanisms underlying observed phenotypes, for example molecular abundances or morphological modifications.

In this work, we will attempt to solve an inverse problem which arose in the study of a signalling pathway. Compared to pathways of metabolic reactions, which are of a limited size comprising up to a few hundreds of proteins, signalling processes involve about 20% of the genome, i.e. thousands of expressed proteins [10], most still unidentified and of unknown function. Protein signalling networks spread information throughout the cell and mediate a number of fundamental processes [11-14]. The growing availability of reliable genomic and proteomic data, made it possible to build up protein interaction maps (PIMs) of increasing complexity. New high-throughput experimental and in silico technologies allow us to monitor protein-protein and genetic interactions: DNA and protein microarrays [15-17], two-hybrid systems [18-20], protein tagging techniques coupled with Mass Spectrometry [21,22], phage display [23,24]. In silico methods also allow us to describe protein-protein (p-p hereafter) interactions or the function of yet unclassified proteins: new p-p interactions might be found on the base of genomic sequence [25,26], using data mining methodologies [27,28], or predicting the composition of protein complexes [29]. In this respect it is worth mentioning a simple though successful method to detect new protein-protein interactions by a comparative genomic analysis of phylogenetic profiles: this approach is based on the assumption that interacting genes tend to co-evolve in different organisms [30,31]. Protein's function can be predicted not only by sequence homology, but also on the basis of their relationships with other proteins whose role is already experimentally assessed [32,33] or by orthology [34]. In order to model the time evolution of a signalling pathway it is necessary to know:

• The species involved in molecular interactions, including chemical reactions

• How the interactions connect the chemical actors and form a signalling network

• How these interactions can be modelled

• The model parameters necessary to computationally simulate the time behaviour of the system.

The mathematical form of the chemical interactions, the model parameters and even the network topology are often only partially known. This implies that model approximations and numerical estimates and, whenever possible, additional specific experimental measurements, are necessary to make a numerical simulation feasible and reliable. This is true whatever modelling techniques is used, such as differential equations [35,36], cellular automata [37], Petri Nets [38] or other hybrid methods [39]. When creating a new model, before starting with numerical procedures, it is necessary to make a survey on all published kinetic data. These data may be found directly in the journal articles, which requires a thorough mining of the literature, or on in annotated databases, collecting and structuring information on p-p interactions.

Only at the end of this phase, further experimental activity and the techniques for parameter's estimate come into play: wherever possible, purposely designed experiments should be carried out in order to directly measure unknown kinetic parameters or to use these measures as constraints for the estimate's algorithm or to decide between alternative models. If new experiments cannot be done, the parameter estimate must rely just on literature data.

## Databases of protein interactions

Protein interactions maps, partially stored in public databases, contain mainly qualitative information on the connectivity of intracellular p-p interactions, while quantitative data on the kinetics of interactions and reactions are still largely unavailable, except for enzyme kinetics. There are to date a number of public databases sites containing qualitative data on protein interaction maps:

• **iHOP**: genetic and protein interactions are extracted by text mining of literature abstract [40,28]

• **Amaze**: it is built upon a complex object-oriented data model that allows it to represent and analyze molecular interactions and cellular processes, kinetic data can potentially be inserted into the data structure [41,42]

• **IntAct**: this offers a database and analysis tools for protein interactions [43,44]

• **Kegg**: it is a large database that contains also several signalling pathways [45,46]

• **DIP**: it contains interactions from over 100 organisms [47,48]

• **IMEx**: it is a consortium of major public providers of molecular interaction data, current members are DIP, IntAct, MINT, MPact, BioGRID, BIND [49]

• **Reactome**: this is a curated database of biological pathways in human beings [50,51]

It should be remarked that a great care has to be payed when dealing with qualitative data: they are often dependent on specific experimental conditions and most of them obtained in unicellular organisms. A straightforward extrapolation of these data to higher organisms is often quite unreliable [52]. Moreover, p-p interactions data in molecular networks are usually obtained from large scale or high-throughput experiments, where spurious interactions are very likely to be collected; computational validation techniques are thus needed to prune primary datasets [53,54]. The same holds when one tries to translate genetic interactions into the corresponding p-p interactions: the two networks have quite different topological properties [55].

The situation is even worse when one analyzes quantitative p-p interactions data in public repositories: the total amount of experimentally-derived kinetic data is only a small percentage of what would be needed to characterize the topology data (i.e. the p-p interactions map). Furthermore, available kinetic constants are often extracted from a single publication where they were measured in vitro, while the kinetics of interactions is highly dependent on experimental set-up and environmental conditions, such as PH, temperature, concentration of other proteins in the cellular environment. It is always advisable to assume that the measured quantities indicate more realistically ranges rather than precise values and care must be used to insert these values into large-scale network models [56]. Nevertheless some investigation of biochemical reactions can anyway be carried out by taking into account the uncertainty of kinetic data [57,58] and by using approximations where some values are missing [39].

This point, however, is already a major concern of the Systems Biology: several programs are being performed aimed at producing sets of validated data, homogeneously refered at specific organisms in well defined and standardized thermo-chemical conditions. The standardization of experimental data sets and of experimental models is the object of an intense debate in the Systems Biology community. There is a wide consensus on the need of standards but also on some drawbacks for a general use of standards as the best research framework in any case. Anyway the way towards a deeper and deeper though slow integration of existing datasets, modelling languages and methodologies appears to be set, as witnessed for example by the wider and wider use of SBML as a language to describe biochemical models, or by the integration of previously separated datasets into a single larger database compliant with new criteria established by international consortia. One example of the latter case is the HUPO – PSI initiative [59], aimed at establishing a common format to represent protein-protein interactions and to synchronize all the already existing databases, as it happened for the genome data: MINT, DIP, BIND and IntAct (see below) already implemented the PSI standard to publish molecular interactions.

p-p interactions in signalling pathways can be divided into two main categories: (a) binding interactions that involve no chemical modifications and (2) biochemical processing, such as phosphorylation and phosphatization. On one hand, the few public sources of kinetic data on binding protein interaction often provide only dissociation constants, i.e. values describing an equilibrium state that offer only partial information about the dynamics of the reaction. To our knowledge, only the KDBI database [60,61] was specifically created to store binding and dissociation rate constants. Other repositories, such as MINT [62,63] and BIND [64,65] offer few examples of dissociation constants. On the other hand, biochemical modifications occur in enzymatic reactions, therefore kinetic data can be found in databases entirely devoted to enzymes, first of all Brenda [66,67] where kinetic constants are specified for several organic substrates, and partially the above cited KDBI.

A further source of signalling pathways and of p-p interactions data, including the kinetic part, are the repositories of biochemical models, though in these models not all the kinetic parameters were measured experimentally and some of them had to be numerically estimated. Among them:

• **Biomodels.Net**: it has been published very recently and it is currently the most curated database of biochemical models, offering tested and verified models in several standard formats included, SBML, CellML and XML [68,69]. A standard for model annotation and curation of biological models called MIRIAM has been recently proposed [70];

• **JWS Online**: another curated repository of models in SBML and PySces formats [71,72]. JWS creators are among the main contributors to the new Biomodels.Net databases and to the MIRIAM initiative;

• **CellML**: repository of biochemicals models in CellML format [73,74]. The CellML team contributes to the MIRIAM project;

• **DOQCS**: this is a large repository of signalling pathways, where all the reactions and kinetic parameters are directly shown, furthermore the models can be downloaded in the Genesis language [75,76]. Also DOQCS curators contributed to the MIRIAM project;

• **ModelDB**: this is a repository of detailed biochemical and electrphysiological processes in the neuronal cell: the models are written in the Genesis language and Neuron languages [77,78].

## Experimental measures of kinetic parameters

The measure of protein activation level is of paramount importance to monitor signalling processes. Several methods exist to quantitate the concentration of protein species, such as immunoblotting, ELISA, radioimmunoassay, protein arrays. If a cellular system is sampled several times over the duration of a given signalling process, a time series can be composed describing the time course of a concentration, for example that of a phosphorilated protein. Radioimmunoassays are very sensitive methods but are even complex, expensive and dangerous to set up; protein arrays offer the advantage of a high throughput approach, while ELISA and immunoblotting are easier to implement and, thus, widely used, though they allow a lower threshold of detection when a very low concentrations of radioactive compounds is present [79]. The experimental error of quantitative immunoblotting can be significantly reduced by computational techniques of data analysis, error estimate and simulation: these allow to monitor activated signalling pathways in real time and to discriminate between different models.

Enzymatic reactions can be monitored, nowadays, in a high throughput scale both in vivo and in vitro: this allows us to measure kinetic parameters characterizing fundamental steps in signalling pathways, such as binding and removal of phosphate groups by kinases and phosphatases. Bioreactors are widely used to perform enzymatic reactions and other biochemical processes but their use for a real time monitoring of products is limited by the sampling process. More recent modified reactors allow a real-time sampling of multiple reactions in vivo over a short reaction time: the reaction broth flows at constant velocity along a thin pipe where spilling at uniform space intervals corresponds to uniform time sampling. In this system the samples can be rapidly quenched and analyzed by mass-spectrometry techniques [80]. Also arrays of nanolitre wells can be used to follow the time course of multiple enzymatic process by the use of optical techniques such as fluorescence and bioluminescence [81]. The analysis of reaction mixtures by mass spectrometry methods makes the use of chromophores and radiolabelling unnecessary, since even the addition of a phosphate group to a large protein can be detected as a precise mass shift in the spectra. In vitro multiplexed assays can be performed on protein chips that are then directly analyzed by surface-enhanced laser desorption/ionization time-of-flight mass spectrometry (SELDI-TOF MS) to monitor enzyme activities [82]. Alternatively complex protein mixtures can be immobilized on micro-beads, where the enzymatic reactions can take place and be monitored by MALDI mass spectrometry [83]. A more difficult issue is to measure kinetic parameters describing binding of proteins without chemical processing, such as ligand-receptor interactions or the formation of protein complexes. Two techniques allow us to calculate kinetic rate constants of binding an unbinding by fitting measured response curves. The Surface Plasmon Resonance (SPR) allows us to measure kinetic constants in vitro in a label-free environment. One of the reactants is immobilized on the sensor surface usually coated with a thin gold film, while the other is free in solution: the behaviour of a polarized light beam hitting the surface in conditions of total internal reflection depends on the refractive index of the surface, that in turn depends on the binding state of the reactants. In essence the SPR measures the angle or the wavelength of the reflected light at which a resonance takes place between the light and the metal electrons: whose changes correspond to the amount of bound molecules. The SPR is already used for high-throughput measurements directly on protein arrays [84-86]. Using a completely different approach called fluorescence cross-correlation spectroscopy (FCS) the kinetics of binding can be quantified directly in living cells. Fluctuations of fluorescence signals can be detected in a very reduced volume, less than a femtolitre, and using a very low fluorophore concentration, up to 5 nM i.e. around 3 molecules/femtolitre, by the use of a tightly focused laser beam. The investigation of the autocorrelation function of the fluorescence signal provides information about the reaction kinetics, the diffusion rates and the equilibrium state. With FCS it is feasible to study at a single molecule level a ligand-receptor interaction with no need of any isotope labeling [87,88].

## "In silico" parameter's estimate

When only a few kinetic parameters are available to implement a model of a signaling network, one might resort to attempting a "theoretical" estimate of these values. The attempt could be performed, in principle, by using an "inverse problem" approach, i.e. by optimizing the unknown parameters of a reaction's model in order to obtain the best possible agreement between simulated and experimental data.

This is the aim of the present work. We devise a methodological workflow (and the corresponding numerical and computational tools) to estimate the unknown reaction constants of a model signalling pathway by starting from (a) a given set of known data of reaction constants and (b) experimental results of the time course of some biochemical species involved in the reaction.

An intracellular signal transduction pathway in the neuronal cell was used as a model system to implement the proposed parameter's estimate procedure.

**Figure 1:**
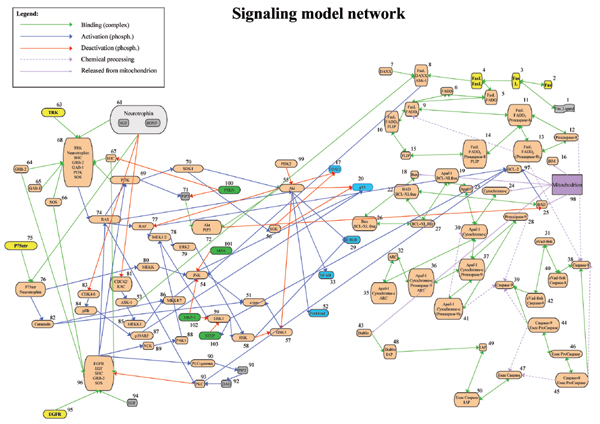
**Scheme of a model signaling network**. Scheme of the signaling network used to demonstrate the validity of the parameter estimate method. The network consists of a series of proteins (the nodes) linked by different types of unary, binary or multiple molecular interactions (shown as the edges of the network). The role of the mitochondrion (in purple) is taken into account. Binding protein-protein interactions are shown by green edges between the nodes, activation and deactivation interactions are in blue and red, respectively, chemical transformations are shown by purple dotted lines, while the release of proteins from the mitochondria in shown in solid purple lines. The signaling process can be activated by the binding of ligands (in grey) to receptors. Every compound is identified by a name and a numerical code.

The chosen pathway is a protein network downstream of the neurotrophic receptors Trk and P75 [89], the Fas receptor regulating an apoptotic cascade [35], the EGF receptor expressed in the CNS [90,91] and in PC12 cells [92,93]. The network structure is based on current literature. The pathway can be divided into two main interconnected sub-systems: an apoptosis pathway and a neurotrophic receptors activated pathway. Neuronal apoptosis can be initiated in three different manners, all leading to the activation of executioner caspases, the effectors of the apoptotic process that kill the cell by irreversible proteolysis of critical cellular constituents: survival factor withdrawal, stress factors and receptor mediated signaling cascade [94,35,95]. In this model the survival factor withdrawal is taken into account by the connections between the two sub-networks, the apoptotic and the neurotrophic driven one, which includes the TRK, EGFR and P75NTR receptors the stress factors are also considered by the presence of a mitochondrion acting as a synthesis machinery for pro-apoptotic proteins (Fig. 1). The signaling pathways forming the network can be activated in several ways; in our model, we chose to trigger the signalling process by the activation of the receptors upstream of the pathways as a consequence of the binding of specific ligands.

The p-p interactions, such as molecular binding, phosphorylation/dephospshorylation or chemical transformations, are described using first order non-linear ordinary differential equations, which take into account also synthesis and degradation processes. The space variable is neglected in this model, since proteins are considered to be close enough to justify the approximation of a geometrical point. The release from the mitochondria was considered to be mathematically equivalent to an additional protein synthesis [94,35]. In this model gene transcription was neglected, owing to the time scale chosen to simulate the temporal evolution of the system, within 60 minutes time. Reactions are treated as a one-step process. For binary activation and inactivation reactions, the following second order kinetics scheme was used, where protein A activates protein B:



The activation rate of protein B is : *v*_*act *_= *K*_*act *_[*A*][*B*]. In the case of binding reactions, resulting in the association/dissociation of protein complexes, the following one-step reaction scheme was used, resulting in a *p*^*th*^-order kinetics, where *p *equals the number of components of the protein complex *C*_*i*_, with forward and reverse rate constants *K *and *K*^-1 ^respectively:



Thus the association rate is *v*_*ass *_= *K *[*C*_1_] [*C*_2_]...[*C*_*n*_] and the dissociation rate is *v*_*diss *_= *K*_-1_[*P*].

Each of the N = 98 nodes of the network is described by the two independent variables *P*_*i *_and *x*_*i *_(i = l...N): the first refers to the total concentration of the protein species, the second to the concentration of the active fraction of that species. Each protein species *i *will thus follow a time evolution given by two coupled reactions:





where *v*_*prod*, *a*_(*v*_*cons*,*a*_), with a = *x*_*i*_, *P*_*i*_, represent production (consumption) reactions having the a-species as object. The complete system of equations describing the system assumes the following explicit mathematical structure:





*N *in the number of nodes, *NP*_*i*,*j *_is the number of different interactions involving the nodes *i *and *j, NC*_*i*,*j*,*r *_is the number of components when *i *is a protein in complex with protein *j *and the  represent the different rate constants. The *r *index accounts for different interactions between nodes *i *and *j*, when existing. The zero-th order terms  and  include the protein synthesis and the release from the mitochondria processes, the linear terms include the protein degradation, chemical autoprocessing and protein complex dissociation; the quadratic terms take into account the activation and de-activation of protein *P_i_*, the polinomial terms describe the protein association into larger complexes. No mass conservation constraint has been imposed to the system.

In our approximation we considered both the topology of the protein interaction map and the kinetic parameters as constant in time, i.e. each protein keeps the same neighbours during the time evolution of the system and interacts with them with constant strength. We decided to completely assign the connectivity matrix of the network on the basis of the existing experimental data. On the other hand, the kinetic parameters were largely unknown on the basis on the same information sources: as a consequence, in this application, the object of the "inverse problem" are the unknown model's constants. The "inverse problem" has been implemented with the following scheme:

1. eqs.(5–6) are solved and the time course of variables *P*_*i *_and *x*_*i *_(i = l...N) are calculated for a given set of model's parameters

2. the predicted time course of certain quantities is compared with the corresponding experimental data and a specific "distance" between time-courses evaluated

3. procedure is iterated up to minimizing that distance

Although, at least in principle, the strategy is simple, in practice the space of parameters to be estimated is very large, thus the strategy of points (1–3) above must rely on the availability of an efficient optimization algorithm. We have resorted to choose Genetic Algorithms (GA) for a number of reasons which will be highlighted in the following section.

## GA: generality, numerical and computational implementation

The genetic algorithm (GA) is a programming technique that mimics biological evolution as a problem-solving strategy. Given a specific problem, the input to the GA is a set (called a "population") of potential solutions (called "individuals") to that problem. Each individual contains a "genome" able to provide a sub-obtimal solution to the problem. This ability could be quantified if a specific fitness function is defined, able to quantify how much an individual, by means of its genome, is fit for the solution of the optimization problem (i.e. to measure the "distance" between the sub-optimal and the optimal solution). The purpose of the GA is to produce successive population of individuals which are generated with the aim of increasing, as much as possible, the fitness of their individuals, i.e. their ability to solve the optimization problem by decreasing that "distance". This is done by producing successive populations of individuals by using the same procedures of the natural selection: mating and mutation. In the GA workflow, given an initial population of individuals, these are evaluated and classified according to their fitness. A selection rule is then defined to allow mating of couples of individuals, that mix their genomes, to form new ones (a further population) and an appropriate frequency of mutation of the genomes is defined, to introduce "new tracts" into individuals (which, in turn, would have been composed only by tracts coming from previous populations). If selection rules for mating and frequency of mutation are appropriatly chosen, the GA produces successive sets of individuals (" generations") which are progressively more and more fit to the optimization problem. In other words, individuals are better and better approximation of the optimal problem's solution.

The " inverse problem" we have attempted to solve starts from the description of a signalling network in terms of biochemical interacting species and reaction's constants. After a mining procedure to discover the value of the known reaction's constants, the system of eqs. (5–6) can be solved, by setting, for the unknown reaction's constants, an initial gauge of values. The solution of eqs.(5–6), in terms of functions describing the predicted time course of each of the system's variables (i.e. the concentration of all the biochemical species of the network), is thus strictly related to the intial set of reaction's constants. If one defines, as individual of the GA, the complete set of reaction's constants (the *l *known constants and the *N *- *l *unknown constants), its ability to produce an optimal solution to the problem can be measured by evaluating the " distance" between the predicted time-course (*f*_*pred*_) of some variables and that effectively measured by an experimental test of the same variables on that network (*f*_*exp*_). Formally, a distance between the two functions representing the *j*-th variable can be defined as follows:



where *t *is the (discrete) time length of the trajectories spanned by the variables. If one has *k *experimentally measured variables, the overall distance between that solution and the "optimal" solution would be



Eq.(8) can be thus retained as the "fitness" function of the considered individual; one can thus measure its "distance" from the "optimal" solution. Indeed, a more general formulation of the fitness function could be given by attributing "empirical" weight factors *α *to each variable, as to produce a different impact on the overall *d *value



**Figure 2:**
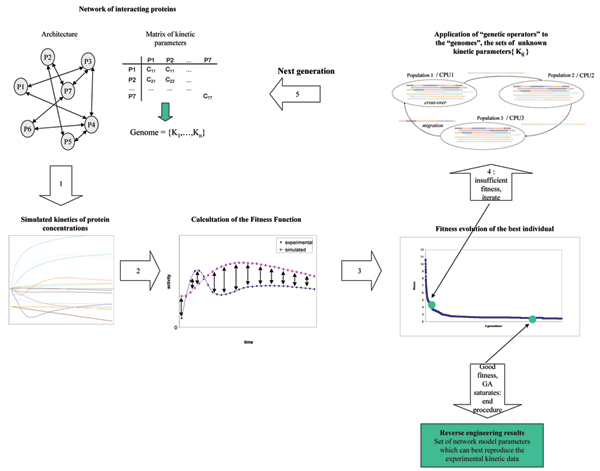
**Genetic algoritm scheme**. Flow chart of the estimate procedure using the genetic algorithm (GA). Every unknown model parameter is called a " gene", while the whole set of parameters to be estimated is defined as the " genome". Every genome is contained within an " individual", the computational entity able to " evolve". An ensemble of genomes corresponds to a "population". The GA procedure begins with an initial random guess of the parameters values used to run a simulation of the model network. This first step is iterated for all the individuals belonging to different populations. For each individual, the simulated time course of the concentrations for specific proteins are compared with the experimental measures and the distances between the functions are calculated. Every individual is thus related to a fitness index, measuring the degree of compatibility of the genome with the experimental constraints. A small number of individuals are selected based on their fitness but also on probabilistic rules: they will have the genomes randomly mutated by genetic operators, giving birth to a new offspring that enters the next generation. At each round the plot describing the evolution of the best fitness computed until then is updated: when it clearly saturates the algorithm stops and the genome corresponding to that fitness is the solution of the algorithm.

The aim of the GA is to produce solutions which progressively reduce the value of the distance of its individuals. The scheme of producing successive "generations" of individuals can be resumed as follows:

1. start with a set of initial *N *individuals {*K*_*i*_, *i *= 1, *n*}, each consisting of the same *l *known constants and by a number *n *- *l *of randomly selected guesses of the unknown constants (Fig. 2). Each value of *K*_*i *_is a real number in the interval [10^-5^,10^0^]. The interval was chosen on the basis of a reasonable number of kinetic values of protein-protein interactions published in the literature

2. for each individual, evaluate the distance **d **of eq.(8)

3. select, according to some defined rule, the individuals to be mated to form the new generation of individuals.

4. perform the mating procedure as follows: given two different individuals {*K*^*A*^(*i*)} and {*K*^*B*^(*i*)}, we randomly select the index *m *(*l *<*m*) and join the two individuals to produce a new individual {*K*^*A*^(*i + *1)} such as



The parameter estimate does not include the topology of the network, that is the connectivity matrix is considered as a constant of the system and no interaction parameter is allowed to go to zero during the optimization procedure. The experimental data used as model constraints to optimize the system are the experimental time course of the concentrations of the active fraction of ERK-1, c-Raf, MEK, PKC-iota proteins [96-98]. These data were obtained measuring the phosphorylation level of these proteins by optical methods, following delivery of NGF to the cell. They are used to calculate the Fitness Function, upon which the GA is based. The optical signal were sampled every 2 minutes for 1 hour, that is a total of 30 sampling points for each protein were used to fit the system. The whole set of model parameters includes 278 indipendent values, of which 15 were extracted from the literature and the other were fitted using the GA.

The algorithm starts assigning every individual with random genome. The initial genes *g*_*i *_were randomly generated in the following manner: *g*_*i*_= 10^α ^where *α *comes from a flat (white noise) distribution in the range *α ∈ *[-5, 0]. This guarantees that the distribution of the initial parameters is flat in the logarithmic scale. The range is expanded in proportion to the number of components NC where the node (i) is a protein complex and if NC > 2: the range becomes *α ∈ *[-5 * (*NC *- 2), 0].

The Fitness Function F() is here defined, for each individual, as the inverse of the squared Euclidean distance between the experimental time course of the concentration of the activated fraction of ERK-1, c-Raf, MEK, PKC-iota proteins (see above) and the simulated time course for the same species, obtained using the genome {*K*_1_,...,*K*_*n*_} of the individual (Fig. 2, step 2) as parameters set; this distance is evaluated across the whole time interval (60 minutes), with a sampling time of 2 minutes:



Here p = *p*_1_*...p*_*np *_indexes the protein species used for the fitness evaluation, t = *t*_1_*...t*_*nt *_indexes the sampling times,  (*t*) and (*t*) are the experimental and simulated time course of the concentration of the activated fraction for protein species p. The fittest genomes, those with the largest value of the Fitness Function, are given a greater probability to be selected to give birth to the next generation of individuals. The probability *Pi *of selection of the *i*^*th *^individual of the population is calculated as:

*S*_*i *_= *F*^1/*t*^, *i *∈ {*population*}      (12)



where 10^-4 ^<*T *< 1 is a constant parameter, used to shape the distribution of the probabilities. It is worth underlining here that the best individuals tend to be selected at each generation, but the probability distribution gives any individual at least a small chance of being selected. The best individuals, then recombine their genes by the cross-over (Fig. 2, step 4), exchanging randomly selected but corresponding segments of the genomes, and eventually the offspring form the next generation (Fig. 2, step 5). The genes of the offspring are also allowed to randomly mutate with a low probability 0.005 <*P*_*mut *_< 0.04. The individuals are distributed among NSp sub-populations, each containing NI of individuals, in our case NI = 16 and 7 < NSp < 33. The evolution process takes place independently within each sub-population at each generation. Every NM generations, with NM of the order of the sub-population size, MI of the best individuals in each population, again selected according to a probabilistic rule, move into a different sub-population, there replacing others that on their turn entered another sub-population: MI is of the order of 10%-30% of NI. This "migration" operator allows a sub-population to partially renew its genetic pool and tends to fasten the evolution process. The algorithm keeps in memory the "optimal" genome and the corresponding fitness, that is the best individual out of all the sub-populations obtained until that stage in the evolution process: these are compared with the best genome and corresponding fitness in the current generation: if the new fitness is better the optimal genome is replaced by the new one. The plot of the optimal fitness versus the generation number describes a monotone non-increasing function: when the curve derivative saturates, the procedure comes to an end and the individual corresponding to the optimal fitness provides the solution genome.

The GA in intrinsically parallel, thus the necessary computation can be very efficiently distributed over several CPUs. The GA was implemented on a cluster of Alpha CPUs, using the Fortran 90 language and the MPI protocol, under Linux operating system. In this implementation each computational node stores the genomes of a single sub-population, which evolves independently, except when there is a migration of individuals. In that case genome vectors are exchanged between the nodes (Fig. 2, step 4). In order to optimize the distribution of the computational load, data communications were reduced, which was exactly compatible with rarely occurring genome migrations.

## Results and Discussion

### Results

The system under investigation does not guarantee that the inverse problem has one unique solution, using the chosen experimental constraints. Therefore we must assume that the GA will find not one single solution but one ensemble of solutions, formed by many sets of model parameters {*K*_1_,...,*K*_*n*_}. The ensemble describes a small sub-space within the entire space of parameters. We decided to sample this sub-space to study the properties of the solutions. The first step in this work was to obtain several numerical estimates of the set of unknown kinetic parameters. The second step was the analysis of the properties of a single solution, then the analysis of the collective properties of the ensemble. Eventually one solution was used as the best estimate of the kinetic parameters, to compare the simulated behaviour of the network with independent experimental data, to assess the reliability of the method. The genetic algorithm was started using each time different random genomes.

**Figure 3:**
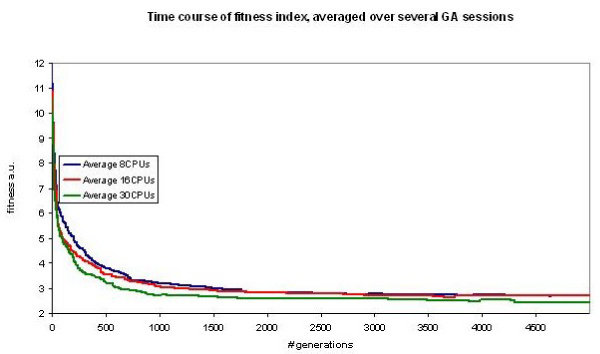
**Fitness index**. Time evolution of the fitness index, during the calculation of the optimal sets of kinetic parameters. The diagrams describes the fitness evolution of the optimal individuals as a result of parallel calculations on 8, 16 and 30 CPUs and are the average over different session, this explains the small discontinuities in the decreasing trend. The time required to reach the saturation decreases as the number of CPUs increases.

The time evolution of the fitness belonging to the optimal individual is a non-increasing function, with an envelope following a decreasing exponential like shape (Fig. 3)

**Figure 4:**
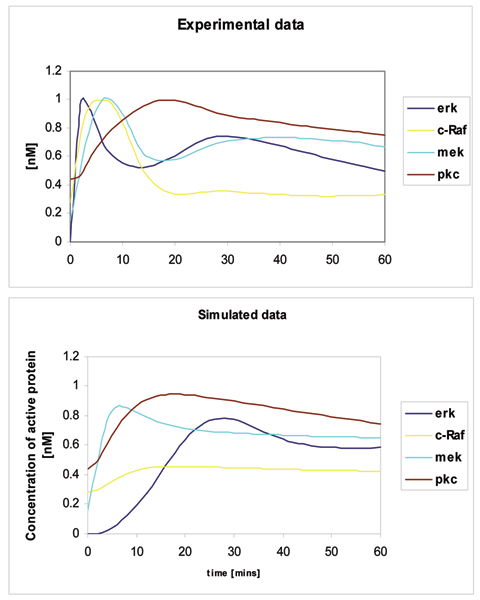
**Simulated and experimental data**. Comparison of experimental and simulated data. The experimental time courses of concentration for the proteins used as constraints in the calculation of the fitness function, is compared with the corresponding simulated behaviours.

When the time derivative approaches zero, the algorithm ends and the current optimal individual is considered to be the estimated solution of the problem. The time of computation necessary to reach a good level of approximation decreases with the number of used CPUs, as it is shown in Fig. 3. This is reasonable since the larger the number of computational nodes in the parallel implementation, the larger the whole population and the probability of selecting a fit individual within a smaller amount of generations. The calculation of the fitness index includes different terms and does not describe in details how similar the simulated and experimental behaviour become as the genetic algorithm proceeds in increasing the fitness of the best individual. Therefore at the end of the algorithmic computation, for each solution the time evolution of the concentration for the proteins chosen as experimental constraints were visually compared to the corresponding simulated behaviour, as shown in the example of Fig. 4, in order to discard meaningless solutions.

Though the experimental and simulated data may appear different, nevertheless, the essential dynamical features, some transients and the following relaxation of the system, are approximately described by the simulation. Since no further significant improvements of the best parameter sets could be obtained using the genetic algorithm, we can attribute the differences to the incomplete connectiveness of the model network, which make some protein concentrations unable to be sufficiently modulated by the activity of the rest of the network. This does not imply that this algorithm proves to be unfit for estimating important properties of the unknown parameters of the model. We obtained a total of 36 solutions of the inverse problem, each of them requiring few days of computation to be calculated.

**Figure 5:**
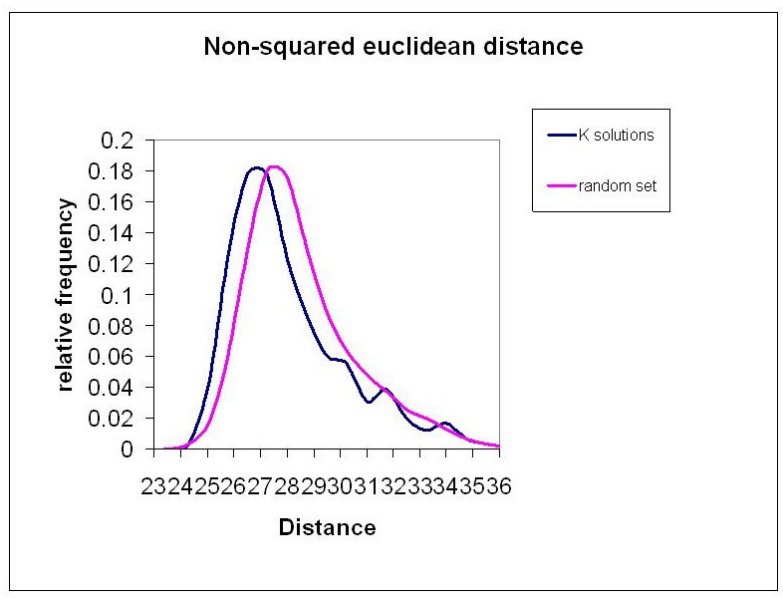
**Proximity matrix of solutions**. Normalized frequency histogram of the elements of the proximity matrix built by computing the non-squared Euclidean distance ||log_10 _*K*_*i*_, log_10 _*K*_*j*_||_*i*, *j *= 1...*n*_, where {*K*_*i*_} and {*K*_*j*_} represent single solution parameter sets and n is the number of unknown model parameters. On the abscissa the distance values. For comparison we show also the distribution of the proximity matrix for a large set of randomly generated K vectors.

The initial random parameter sets were completely altered by the genetic operators, both by the cross-over and the random mutation, which affected at least once every element of the genomes, therefore the final outcome of the algorithm, the optimal genome, has lost every numerical similarity with the initial parameter sets. These two points together could have two kind of consequences: either all the the reactions are necessary for a correct dynamics of the network, or only few reactions dominate the dynamics and guarantee that the chosen experimental constraints are satisfied, while the other rate constants may just fluctuate almost randomly. Further analysis, later in this article, will show that the second hypothesis is probably the correct one. Some more hints come from the calculation of the proximity matrix of the logarithm of solution vectors, whose elements are the non-squared Euclidean distances between all the couples of solutions genomes. We have plotted the frequency distribution of the elements (Fig. 5) and compared it to a the distribution of a large ensemble of random vectors, generated using the same criteria and value ranges as the initial genomes in first step of the genetic algorithm.

**Figure 6:**
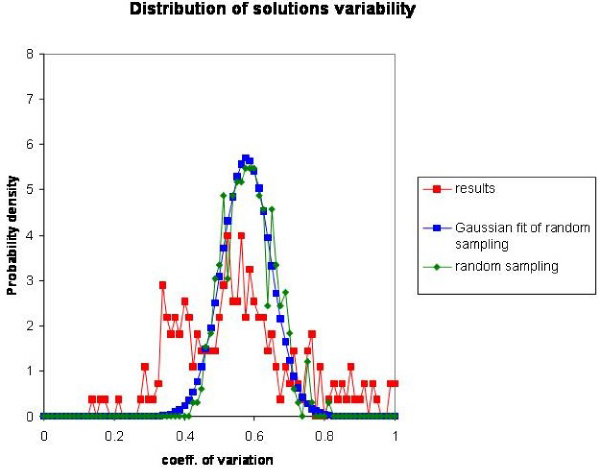
**Distribution of the coefficients of variation of solution parameters. **The coefficient of variation *StdDev*(log_10_*K*_*i*_)*/Mean*(log_10_*K*_*i*_), where {K_*i*_}_*i *= 1...*n *_is any kinetic parameter, was computed for every parameter across the entire ensemble of solution sets. Their distribution is shown (red line). For comparison the distribution of the coefficient of variation of a variable X is shown (green line), where X is sampled from a uniform distribution in the interval [-5,0]. The distribution of the coefficient of variation can be approximated by a Gaussian density function N(*μ, σ*) with *μ *= 2/ and *σ *= 0.07 (in blue): the *μ *value is the coefficient of variation of the uniform distribution, while *σ *is the standard deviation of a random set of coefficients of variation obtained by sampling the uniform distribution in the interval [-5,0] (Fig. 7). A coefficient of variation smaller than 0.33 has a probability of random occurrence ≤ 0.0002, while the 17 parameters selected using the genetic algorithm represent 6.5% of the whole (Fig. 7), that is a coefficient of variation smaller the 0.33 have a proability of occurrence of 0.065 in the solution set.

**Figure 7:**
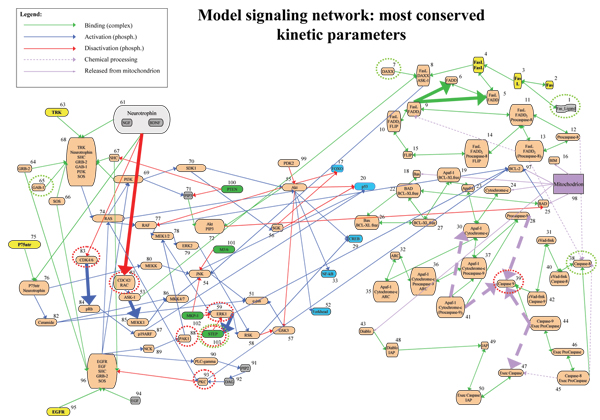
**Variability of solutions**. Most conserved kinetic parameters. The coefficient of variation *StdDev*(log_10_*K*_*i*_)*/Mean*(log_10_*K*_*i*_), where {*K*_*i*_}_*i *= 1...*n *_is any kinetic parameter, was computed for every parameter across the entire ensemble of solution sets. The kinetic parameters with a ratio ≤ 0.33 are highlighted in the network graphical representation: thick arrows refer to kinetic rates of protein-protein interaction, the red circles refer to degradation rates and the green circles to synthesis rates.

The asymmetrical bell shape is typical of the distributions of the distances between all the geometrical points contained in a generic hypercube, here described by the parameter ranges in the n-dimensional space, where n = number of unknown parameters: for instance the same distribution pattern holds even in two dimensions. The two distributions have very similar shapes, though the solutions are slightly shifted towards shorter distances, a feature that is not surprising since the solutions belong to a smaller sub-space of the cited n-dimensional hypercube, thus the corresponding points in the parameter space are closer one to the other. The fact that the distribution of solutions is shifted of a small value, about 20% of the bell width, suggests that probably only few parameters contribute to this shift while the others are essentially randomly distributed. After analyzing the solutions parameter sets as static entities, separated from the network dynamics they describe, they must eventually be characterized on the basis of such dynamics. To make again a genetic comparison, it is not sufficient to analyze the "genotypes", the solutions, but rather the corresponding "phenotypes", the time course of protein concentrations. Each of solution parameter sets can be used to simulate the signal transduction process in the network, since it is considered to be a "realistic" set of kinetic parameters. The dynamics described by each of the solutions is slightly different, though, in any case, the time course of protein concentrations meets the experimental constraints used for the genetic algorithm. This similarity can be explained by a closer investigation of the detailed structure of such ensemble, to understand what explains the similarities and, at the same time, the differences among the simulated dynamics obtained with the different estimated solutions. We computed the ratio, in the logarithmic scale, between the standard deviation and mean for each parameter *K*_*i *_belonging to the genome, with i = l...N, and across the whole ensemble of computed solutions {Solutions}, that is the vector of coefficients of variation:



where N is the number of parameters. The 17 parameters showing a ratio smaller than 0.3 were considered as conserved elements across the ensemble of solutions. This threshold was chosen on the basis of the distribution of the coefficient of variation of a variable X, where X is sampled from a uniform distribution in the interval [-5,0]. The distribution of the coefficient of variation can be approximated by a Gaussian density function N(*μ, σ*) with *μ *= 2/ and *σ *= 0.07: the *μ *value is the coefficient of variation of the uniform distribution, while *σ *is the standard deviation of a random set of coefficients of variation obtained by sampling the uniform distribution in the interval [-5,0] (Fig. 6). A coefficient of variation smaller than 0.33 has a probability of random occurrence ≤ 0.0002, while the 17 parameters selected using the genetic algorithm represent 6.5% of the whole (Fig. 7), that is a coefficient of variation smaller the 0.33 have a proability of occurrence of 0.065 in the solution set, therefore statistically significant.

The parameters highlighted in fig. 7 correspond to reactions belonging both to he neurotrophic signal transduction pathway and to the apoptosis pathway: the Caspase-8 and Caspase-9, final mediators of the apoptotic process, are involved in this subgroup of reactions, as well as the PKC protein, one of the " bridges" in this model between the two main pathways of the network, other reactions belong to the NGF-TRK signal transduction process. This group of reactions spans all the typologies included in the model: protein-protein activation/inactivation, protein binding, binary chemical transformations, unary synthesis and degradation rates. The conserved parameter values appear as key elements to guarantee that the experimental data used for the genetic algorithm estimate procedure are met. Furthermore this implies that the same parameters are required for a correct signal transduction, leading to a simulated outcome in agreement to the experimental one.

We have also investigated the level of complexity of the network dynamics through the evaluation of the eigenvalue spectrum and the eigenvectors of the Jacobian matrix of the system of eqs.(5–6). The Jacobian was evalued at a fixed time point (corresponding to t = 60 mins) of a time simulation perfomed by using one parameter set obtained by the GA procedure. The eigenvalue spectrum spans 24 orders of magnitude, from 10^-22 ^to 10^2^, with about 75% of them being real negative values and 25% real positive ones: this implies that the majority of kinetic modes (eigenvectors) in the diagonalized system lead to an exponential decay, though with a large spectrum of decay rates. The components of the orthonormal 2N eigenvectors along the original set of 2N coordinates *x*_*i*_, *P*_*i *_describe how the nodes of the networks are involved in the corresponding kinetic modes. In this respect 20 eigenvectors have significant components (larger than 0.1) just along one of the coordinate, therefore the corresponding dynamics involves essentially only one node of the network, while other 57 eigenvectors have significant components only along two coordinates corresponding to two distinct nodes. On the other hand more than 50% of the eigenvectors have significant components along 3 or more coordinates, up to 12: they thus correspond to more complex modes that involve a large number of network proteins. Moreover many eigenvectors project on the same coordinates, which means that many proteins are inolved in different kinetic modes. In conclusion we can say that a group of small subnetworks exists, composed by one or two nodes, that show a very simple increasing or decreasing dynamics, but this group cannot describe in an exhaustive way the system dynamics: only a complex relation between several kinetic modes can account for the simulated bahaviour.

### Discussion

#### Different methods for parameter estimate and fitting

GA has proven to be a powerful and successful problem-solving strategy. It has been used, in fact, to solve NP-complete optimization problems in a wide variety of fields such as chemistry, biology, engineering, astrophysics, aerospace, electronics, mechanical and electrical design, military plans, mathematics, robotics and many others. Notable examples of GAs applications in molecular biology are in modelling of genetic and regulatory networks [99,7,100], predicting protein structure and evolution [101,102], classification of odorant molecules [103], investigation of the metabolome [104]. We have chosen to estimate the unknown parameters of our signalling network model by minimizing the difference between the simulated output of the model and the corresponding experimental observations. The function to minimize is a vector distance between experimental and simulated concentrations sampled along a time interval; the distance depends on the whole set of model parameters. A number of other numerical methods exist to minimize such multivariate functions: downhill simplex, direction set, conjugate gradient, variable metric, linear programming, simulated annealing (SA) [105,106]. These methods have the common feature of progressively modifying the same function, until a minimum is reached. In particular, the SA is a Monte Carlo non evolutionary strategy based on a thermalization-equivalent process of the system, in fact it is commonly used in computational physics to find minima of the energy states. At the heart of the method is an analogy with the slow cooling of liquids, a process called "annealing". A slow exploration of the energy landscape ensures that the absolute minimum is reached, if it is unique. From another point of view, the SA could also be consider a form of GA where a single individual evolves alone by means of random mutations, without any cross-over as no other individual is available. The SA and another problem-solving technique called the hill-climbing show some similarities to GAs: in both algorithms one single solution is evolving, instead of a population of candidate solutions. These algorithms start with one single random solution: at each round the candidate solution can mutate and its fitness is evaluated: if it is better than the previous one it is kept and passed to the next round, otherwise it is discarded and the previous one is mutated again. In the SA discarding a solution is based also on a specific parameter called the temperature, that gives even unfit solutions a non-zero probability of passing to the next round. In a preliminary phase of our work we compared the computational performances of the GA with the SA to solve the same problem of parameter estimate in the protein network. The SA was implemented in the classical version and run on a single CPU: for the specific inverse problem described in this work, the performances on the GA were better than the SA even on a single computational node, since functional minima were found faster. The existence of multiple solutions did not require explicitly the use of the SA. The GA is inherently parallelizable because of the existence of many populations, each attributable to a different CPU in a multi-processor architecture using the MPI protocol. The heaviest computational task of the GA is by far the evaluation of the Fitness Function, since the dynamics of the network must be simulated for each individual at every generation, while the application of the genetic operators is rather instantaneous, therefore a good solution is to distribute the computation over many CPUs running in parallel. To exploit in the best possible way the computing power, the computational load should be equally distributed among the nodes: this was obtained by assigning to the CPUs populations with uniform size. Furthermore it is recommended to minimize the communications among the nodes, as a consistent data transfer can considerably slow down the performance of the machine: here the data exchange is restricted almost exclusively to the exchange of genomes during the migration, which represents an absolutely negligible amount of transferred data. Thus the nodes act as almost independent entities and the performances of the GA scale approximately inversely with the number of nodes, that is the algorithm requires O(l/Cn) generations to find the solution, where Cn is the number of computational nodes.

#### Comparison of simulations with experimental data and multiple solutions of the inverse problem

We believe anyway that the major limitation of this model is not the degree of approximation used to describe protein-protein interactions but that some other biologically relevant features are missing, such as the connections with the gene transcription network and with other signalling pathways and the role of the space diffusion, which may be the subject of future improvement of the model. These reasons should explain why this network is more a test case for the implementation of the GA in the inverse problems domain than an accurate description of the neurotrophic and apoptotic signal transduction processes. It is likely that other independent experimental data would allow us to have an unambiguous selection among the different solutions of the Pareto set, in two different manners: either these data could be added as additional constraints from the beginning of the GA procedure, to consistently reduce the Pareto set since the beginning, or they could play the role of independent criteria to select one single or at least a subset of proper solutions obtained by the GA procedure as presented in this work. The modelled signaling network must also be able to respond to a variety of external stimuli, coming from the rest of the cellular environment, as a consequence of this the diversity displayed by these behaviours is compatible with the existence of this ability. The lack of functional connections to other signalling pathways does not allow the network to directly display these potential modalities of response. A related point is the robustness of the system. The optimal solutions belonging to the Pareto set correspond to different dynamical evolutions, though all meet the experimental conditions: this suggests that the network shows some robustness since it is able to guarantee the same signal transduction in many different conditions, with very different combinations of protein-protein interactions strength. The robustness is a fundamental property of biological systems, essential for survival when it is necessary to face dangerous situations and sudden changes in the cellular environment.

#### Conserved kinetic parameters

At the end of this work we found out that a sub-vector of the kinetic parameters is characterized by a small coefficient of variation



across the Pareto set of optimal solutions, where *K*_*j *_is a model parameter value describing the *i*^*th*^interaction/reaction. This is an important and informative result since those parameters correspond to protein-protein interactions and synthesis/degradation processes essential to make the model correctly describe the experimental data used as constraints for the parameter estimate procedure. This sub-vector includes protein-protein interactions and single protein reactions that could explain the robustness of the network dynamics, across the whole Pareto set. The sub-vector can be considered as composed by values almost unambiguously estimated, within a reasonable error, compared to the rest of the parameters. The existence of this sub-vector supports the idea that a sufficient amount of experimental determinants could sufficiently condition the inverse problem to allow a reliable estimate of the whole parameter set. What we have done is in fact to sample the space of solutions of the inverse problem using a genetic algorithm: a larger number of experimental constraints would reduce the dimension of the space of solutions.

## Conclusion

In this work we have discussed the problem of mining, measuring and estimating the value of parameters needed in mathematical models describing the signalling processes mediated by protein-protein interactions. The lack of kinetic interaction rates measured in reliable *in vivo *and *in vitro *experiments is currently the major limitation to the creation of complex models of signaling pathway. We have attempted to show that biological information can be also extracted from a model which, levaraging on known kinetic parameters, attempts to provide a qualitative estimate of unknown parameters, even in the case of ill-conditioned optimization problems. We have thus sampled the space of model parameters using the Genetic Algorithm to estimate sets of unknown parameters. This sampling procedure has shown the existence of a basin of attraction for several kinetic constants. This might be interpreted as a a necessary condition for the network to produce a specific outcome of the time – course of its components. The estimated value of some of the parameters have shown a small coefficient of variation across the set of solutions, though the high dimensionality of this space allows to estimate realiable values and draw conlusions only on these few parameters.

## Authors' contributions

The author(s) contributed equally to this work

## References

[B1] Pullan AJ, Buist ML, Sands GB, Cheng LK, Smith NP (2003). Cardiac electrical activity – from heart to body surface and back again. J Electrocardiol.

[B2] Bertrand C, Hamada Y, Kado H (2001). MRI prior computation and parallel tempering algorithm: a probabilistic resolution of the MEG/EEG inverse problem. Brain Topogr.

[B3] Faugeras O, Adde G, Charpiat G, Chefd'hotel C, Clerc M, Deneux T, Deriche R, Hermosillo G, Keriven R, Kornprobst P, Kybic J, Lenglet C, Lopez-Perez L, Papadopoulo T, Pons JP, Segonne F, Thirion B, Tschumperle D, Vieville T, Wotawa N (2004). Variational, geometric, and statistical methods for modeling brain anatomy and function. Neuroimage.

[B4] Chou KG (2005). Progress in protein structural class prediction and its impact to bioinformatics and proteomics. Curr Protein Pept Sci.

[B5] Congreve M, Murray CW, Blundell TL (2005). Structural biology and drug discovery. Drug Discov Today.

[B6] Russell RB, Alber F, Aloy P, Davis FP, Korkin D, Pichaud M, Topf M, Sail A (2004). A structural perspective on protein-protein interactions. Curr Opin Struct Biol.

[B7] Swain M, Hunniford T, Dubitzky W, Mandel J, Palfreyman N (2005). Reverse-engineering gene-regulatory networks using evolutionary algorithms and grid computing. J Clin Monit Comput.

[B8] Tegner J, Yeung MK, Hasty J, Collins JJ (2003). Reverse engineering gene networks: integrating genetic perturbations with dynamical modeling. Proc Natl Acad Sci USA.

[B9] Nam D, Park CH (2000). Multiobjective Simulated Annealing: A Comparative Study to Evolutionary Algorithms. Int J Fuzzy Systems.

[B10] Shaw G (2003). Cracking the Code of Signal Transduction The need is growing for a map of signal transduction that shows how wired and communicative a cell's proteins are. Genom Proteom.

[B11] Bhalla US (2003). Understanding complex signaling networks through models and metaphors. Prog Biophys Mol Biol.

[B12] Meldolesi J, Role L (2001). Signalling mechanisms. Curr Opin Neurobiol.

[B13] Steffen M, Petti A, Aach J, D'Haeseleer P, Church (2002). Automated modelling of signal transduction networks. BMC Bioinformatics.

[B14] Gilman AC, Simon MI, Bourne HR, Harris BA, Long R, Ross EM, Stull JT, Taussig R, Arkin AP, Cobb MH, Cyster JG, Devreotes PN, Ferrell JE, Fruman D, Gold M, Weiss A, Berridge MJ, Cantley LC, Catterall WA, Coughlin SR, Olson EN, Smith TF, Brugge JS, Botstein D, Dixon JE, Hunter T, Lefkowitz RJ, Pawson AJ, Sternberg PW, Varmus H, Subramaniam S, Sinkovits RS, Li J, Mock D, Ning Y, Saunders B, Sternweis PC, Hilgemann D, Scheuermann RH, DeCamp D, Hsueh R, Lin KM, Ni Y, Seaman WE, Simpson PC, O'Connell TD, Roach T, Choi S, Eversole-Cire P, Fraser I, Mumby MC, Zhao Y, Brekken D, Shu H, Meyer T, Chandy G, Heo WD, Liou J, O'Rourke N, Verghese M, Mumby SM, Han H, Brown HA, Forrester JS, Ivanova P, Milne SB, Casey PJ, Harden TK, Doyle J, Gray ML, Michnick S, Schmidt MA, Toner M, Tsien RY, Natarajan M, Ranganathan R, R SG (2002). Overview of the Alliance for Cellular Signaling. Nature.

[B15] Ramachandran N, Larson DN, Stark PR, Hainsworth E, LaBaer J (2005). Emerging tools for real-time label-free detection of interactions on functional protein microarrays. FEBS J.

[B16] Zangar RC, Varnum SM, Bollinger N (2005). Studying cellular processes and detecting disease with protein microarrays. Drug Metab Rev.

[B17] Ross JS, Symmans WF, Pusztai L, Hortobagyi GN (2005). Pharmacogenomics and clinical biomarkers in drug discovery and development. Am J Clin Pathol.

[B18] S F (2005). : High-throughput two-hybrid analysis: the promise and the peril. FEBS J.

[B19] Stelzl U, Worm U, Lalowski M, Haenig C, Brembeck FH, Goehler H, Stroedicke M, Zenkner M, Schoenherr A, Koeppen S, Timm J, Mintzlaff S, Abraham C, Bock N, Kietzmann S, Goedde A, Toksoz E, Droege A, Krobitsch S, Korn B, Birchmeier W, Lehrach H, Wanker EE (2005). A human protein-protein interaction network: a resource for annotating the proteome. Cell.

[B20] Ito T, Chiba T, Ozawa R, Yoshida M, Hattori M, Sakaki Y (2001). A comprehensive two-hybrid analysis to explore the yeast protein interactome. Proc Natl Acad Sci USA.

[B21] Zhou H, Boyle R, Aebersold R (2004). Quantitative protein analysis by solid phase isotope tagging and mass spectrometry. Methods Mol Biol.

[B22] Schneider LV, Hall MP (2005). Stable isotope methods for high-precision proteomics. Drug Discov Today.

[B23] Geuijen CA, Bijl N, Smit RC, Cox F, Throsby M, Visser TJ, Jongeneelen MA, Bakker AB, Kruisbeek AM, Goudsmit J, De Kruif J (2005). A proteomic approach to tumour target identification using phage display, affinity purification and mass spectrometry. Eur J Cancer.

[B24] Stratmann T, Kang AS (2005). Cognate peptide-receptor ligand mapping by directed phage display. Proteome Sci.

[B25] Shi TL, Li YX, Cai YD, Chou KC (2005). Computational methods for protein-protein interaction and their application. Curr Protein Pept Sci.

[B26] Huynen MA, Snel B, von Mering CPB (2003). Function prediction and protein networks. Curr Opin Cell Biol.

[B27] Hoffmann R, Krallinger M, Andres E, Tamames J, Blaschke C, Valencia A (2005). Text mining for metabolic pathways, signaling cascades, and protein networks. Sci STKE.

[B28] Hoffmann R, Valencia A (2004). A gene network for navigating the literature. Nat Genet.

[B29] Bader GD, Hogue CW (2003). An automated method for finding molecular complexes in large protein interaction networks. BMC Bioinformatics.

[B30] Barker D, Pagel M (2005). Predicting functional gene links from phylogenetic-statistical analyses of whole genomes. PLoS Comput Biol.

[B31] Pazos F, Valencia A (2001). Similarity of phylogenetic trees as indicator of protein-protein interaction. Protein Eng.

[B32] Sun S, Zhao Y, Jiao Y, Yin Y, Cai L, Zhang Y, Lu H, Chen R, Bu D (2006). Faster and more accurate global protein function assignment from protein interaction networks using the MFGO algorithm. FEBS Lett.

[B33] Vazquez A, Flammini A, Maritan A, Vespignani A (2003). Global protein function prediction from protein-protein interaction networks. Nat Biotechnol.

[B34] Persico M, Ceol A, Gavrila C, Hoffmann R, Florio A, Cesareni G (2005). HomoMINT: an inferred human network based on orthology mapping of protein interactions discovered in model organisms. BMC Bioinformatics.

[B35] Fussenegger M, Bailey JE, Varner J (2000). A mathematical model of caspase function in apoptosis. Nat Biotechnol.

[B36] Schoeberl B, Eichler-Jonsson C, Gilles ED, Muller G (2002). Computational modeling of the dynamics of the MAP kinase cascade activated by surface and internalized EGF receptors. Nat Biotechnol.

[B37] Caudle RM (2006). Memory in astrocytes: a hypothesis. Theor Biol Med Model.

[B38] Lee DY, Zimmer R, Lee SY, Park S (2006). Colored Petri net modeling and simulation of signal transduction pathways. Metab Eng.

[B39] Bentele M, Lavrik I, Ulrich M, Stosser S, Heermann DW, Kalthoff H, Krammer PH, Eils R (2004). Mathematical modeling reveals threshold mechanism in CD95-induced apoptosis. J Cell Biol.

[B40] iHOP: Information Hyperlinked Over Proteins. http://www.pdg.cnb.uam.es/UniPub/iHOP.

[B41] Amaze workbench. http://www.amaze.ulb.ac.be/lightbench.

[B42] Lemer C, Antezana E, Couche F, Fays F, Santolaria X, Janky R, Deville Y, Richelle J, Wodak SJ (2004). The aMAZE LightBench: a web interface to a relational database of cellular processes. Nucleic Acids Res.

[B43] Intact:molecular interaction database. http://www.ebi.ac.uk/intact/index.jsp..

[B44] Hermjakob H, Montecchi-Palazzi L, Lewington C, Mudali S, Kerrien S, Orchard S, Vingron M, Roechert B, Roepstorff P, Valencia A, Margalit H, Armstrong J, Bairoch A, Cesareni G, Sherman DRA (2004). IntAct: an open source molecular interaction database. Nucleic Acids Res.

[B45] Kegg: Kyoto Encyclopedia of Genes and Genomes. http://www.genome.jp/kegg.

[B46] Kanehisa M, Goto S, Hattori M, Aoki-Kinoshita KF, Itoh M, Kawashima S, Katayama T, Araki M, Hirakawa M (2006). From genomics to chemical genomics: new developments in KEGG. Nucleic Acids Res.

[B47] DIP: Database of Interacting Proteins. http://dip.doe-mbi.ucla.edu.

[B48] Salwinski L, Miller CS, Smith AJ, Pettit FK, Bowie JU, Eisenberg D (2004). The Database of Interacting Proteins: 2004 update. Nucleic Acids Res.

[B49] IMEx: The International Molecular Exchange Consortium. http://imex.sourceforge.net..

[B50] Reactome: database of biological pathways. http://www.genomeknowledge.org.

[B51] Joshi-Tope G, Gillespie M, Vastrik I, D'Eustachio P, Schmidt E, de Bono B, Jassal B, Gopinath G, Wu G, Matthews L, Lewis S, Birney E, Stein L (2005). Reactome: a knowledgebase of biological pathways. Nucleic Acids Res.

[B52] Cesareni G, Ceol A, Gavrila C, Palazzi LM, Persico M, Schneider MV (2005). Comparative interactomics. FEBS Lett.

[B53] Chen J, Hsu W, Lee ML, Ng SK (2005). Discovering reliable protein interactions from high-throughput experimental data using network topology. Artif Intell Med.

[B54] Patil A, Nakamura H (2005). Filtering high-throughput protein-protein interaction data using a combination of genomic features. BMC Bioinformatics.

[B55] Coulomb S, Bauer M, Bernard D, Marsolier-Kergoat MC (2005). Gene essentiality and the topology of protein interaction networks. Proc Biol Sci.

[B56] Famili I, Mahadevan R, Palsson BO (2005). k-Cone analysis: determining all candidate values for kinetic parameters on a network scale. Biophys J.

[B57] Klipp E, Liebermeister W, Wierling C (2004). Inferring dynamic properties of biochemical reaction networks from structural knowledge. Genome Inform Ser Workshop Genome Inform.

[B58] Wang L, Hatzimanikatis V (2006). Metabolic engineering under uncertainty. I: Framework development. Metab Eng.

[B59] HUPO-PSI: Human Proteome Organization – Proteomics Standards Initiative. http://psidev.sourceforge.net.

[B60] KDBI: Kinetic data of Biomolecular Interactions. http://xin.cz3.nus.edu.sg/group/kdbi/kdbi.asp.

[B61] Ji ZL, Chen X, Zhen CJ, Yao LX, Han LY, Yeo WK, Chung PC, Puy HS, Tay YT, Muhammad A, Chen YZ (2003). KDBI: Kinetic Data of Bio-molecular Interactions database. Nucleic Acids Res.

[B62] MINT, Molecular Interations Database. http://mint.bio.uniroma2.it/mint.

[B63] Zanzoni A, Montecchi-Palazzi L, Quondam M, Ausiello G, Helmer-Citterich M, Cesareni G (2002). MINT: a Molecular INTeraction database. FEBS Lett.

[B64] BIND: Biomolecular Interaction Network Database. http://www.bind.ca/Action.

[B65] Gilbert D (2005). Biomolecular Interaction Network Database. Briefings in Bioinformatics.

[B66] Brenda: Enzyme database. http://www.brenda.uni-koeln.de.

[B67] Schomburg I, Chang A, Ebeling C, Gremse M, Heldt C, Huhn G, Schomburg D (2004). BRENDA, the enzyme database: updates and major new developments. Nucleic Acids Res.

[B68] Biomodels.Net. http://www.ebi.ac.uk/biomodels.

[B69] Le Novere N, Finney A, Hucka M, Bhalla US, Campagne F, Collado-Vides J, Crampin EJ, Halstead M, Klipp E, Mendes P, Nielsen P, Sauro H, Shapiro B, Snoep JL, Spence HD, Wanner BL (2005). Minimum information requested in the annotation of biochemical models (MIRIAM). Nat Biotechnol.

[B70] Le Novere N, Bornstein B, Broicher A, Courtot M, Donizelli M, Dharuri H, Li L, Sauro H, Schilstra M, Shapiro B, Snoep JL, Hucka M (2006). BioModels Database: a free, centralized database of curated, published, quantitative kinetic models of biochemical and cellular systems. Nucleic Acids Res.

[B71] JWS Online. http://jjj.biochem.sun.ac.za/index.html.

[B72] Olivier BG, Snoep JL (2004). Web-based kinetic modelling using JWS Online. Bioinformatics.

[B73] CellML. http://www.cellml.org.

[B74] Lloyd CM, Halstead MD, Nielsen PF (2004). CellML: its future, present and past. Prog Biophys Mol Biol.

[B75] DOQCS: Database of Quantitative Cellular Signaling. http://doqcs.ncbs.res.in.

[B76] Sivakumaran S, Hariharaputran S, Mishra J, Bhalla US (2003). The Database of Quantitative Cellular Signaling: management and analysisof chemical kinetic models of signaling networks. Bioinformatics.

[B77] ModelDB. http://senselab.med.yale.edu/senselab/ModelDB/default.asp.

[B78] Hines ML, Morse T, Migliore M, Carnevale NT, Shepherd GM (2004). ModelDB: A Database to Support Computational Neuroscience. J Comput Neurosci.

[B79] Schilling M, Maiwald T, Bohl S, Kollmann M, Kreutz C, Timmer J, Klingmuller U (2005). Computational processing and error reduction strategies for standardized quantitative data in biological networks. Febs J.

[B80] Visser D, van Zuylen GA, van Dam JC, Oudshoorn A, Eman MR, Ras C, van Gulik WM, Frank J, van Dedem GW, Heijnen JJ (2002). Rapid sampling for analysis of in vivo kinetics using the BioScope: a system for continuous-pulse experiments. Biotechnol Bioeng.

[B81] Young IT, Moerman R, Van Den Doel LR, lordanov V, Kroon A, Dietrich HR, Van Dedem GW, Bossche A, Gray BL, Sarro L, Verbeek PW, Van Vliet LJ (2003). Monitoring enzymatic reactions in nanolitre wells. J Microsc.

[B82] Thulasiraman V, Wang Z, Katrekar A, Lomas L, Yip TT (2004). Simultaneous monitoring of multiple kinase activities by SELDI-TOF mass spectrometry. Methods Mol Biol.

[B83] Schluter H, Jankowski J, Rykl J, Thiemann J, Belgardt S, Zidek W, Wittmann B, Pohl T (2003). Detection of protease activities with the mass-spectrometry-assisted enzyme-screening (MES) system. Anal Bioanal Chem.

[B84] Jung SO, Ro HS, Kho BH, Shin YB, Kim MG, Chung BH (2005). Surface plasmon resonance imaging-based protein arrays for high-throughput screening of protein-protein interaction inhibitors. Proteomics.

[B85] Yuk JS, Kim HS, Jung JW, Jung SH, Lee SJ, Kim WJ, Han JA, Kim YM, Ha KS (2006). Analysis of protein interactions on protein arrays by a novel spectral surface plasmon resonance imaging. Biosens Bioelectron.

[B86] Ro HS, Koh BH, Jung SO, Park HK, Shin YB, Kim MG, Chung BH (2006). Surface plasmon resonance imaging protein arrays for analysis of triple protein interactions of HPV, E6, E6AP, and p53. Proteomics.

[B87] Kohl T, Haustein E, Schwille P (2005). Determining protease activity in vivo by fluorescence cross-correlation analysis. Biophys J.

[B88] Pramanik A (2004). Ligand-receptor interactions in live cells by fluorescence correlation spectroscopy. Curr Pharm Biotechnol.

[B89] Barrett GL (2000). The p75 neurotrophin receptor and neuronal apoptosis. Prog Neurobiol.

[B90] Kramer A, Yang FC, Snodgrass P, Li X, Scammell TE, Davis FC, Weitz CJ (2001). Regulation of daily locomotor activity and sleep by hypothalamic EGF receptor signalling. Science.

[B91] Islam R, Wei SY, Chiu WH, Hortsch M, Hsu JC (2003). Neuroglian activates Echinoid to antagonize the Drosophila EGF receptor signaling pathway. Development.

[B92] Gatti A (2003). Divergence in the upstream signaling of nerve growth factor (NGF) and epidermal growth factor (EGF). Neuroreport.

[B93] Vaudry D, Stork PJ, Lazarovici P, Eiden LE (2002). Signaling pathways for PC12 cell differentiation: making the right connections. Science.

[B94] Brunet A, Datta SR, Greenberg ME (2001). Transcription-dependent and -independent control of neuronal survival by the PI3K-Akt signaling pathway. Curr Opin Neurobiol.

[B95] Raoul C, Pettmann B, Henderson CE (2000). Active killing of neurons during development and following stress: a role for p75(NTR) and Fas?. Curr Opin Neurobiol.

[B96] Chevet E, Lemaitre G, Janjic N, Barritault D, Bikfalvi A, Katinka MD (1999). 1999. Fibroblast growth factor receptors participate in the control of mitogen-activated protein kinase activity during nerve growth factor-induced neuronal differentiation of PC12 cells. J Biol Chem.

[B97] Wooten MW, Vandenplas ML, Seibenhener ML, Geetha T, Diaz-Meco MT (2001). Nerve growth factor stimulates multisite tyrosine phosphorylation and activation of the atypical protein kinase C's via a src kinase pathway. Mol Cell Biol.

[B98] Kao S, Jaiswal RK, Kolch W, Landreth GE (2001). Identification of the mechanisms regulating the differential activation of the mapk cascade by epidermal growth factor and nerve growth factor in PC12 cells. J Biol Chem.

[B99] Quayle AP, Bullock S (2006). Modelling the evolution of genetic regulatory networks. J Theor Biol.

[B100] Kikuchi S, Tominaga D, Arita M, Takahashi K, Tomita M (2003). Dynamic modeling of genetic networks using genetic algorithm and S-system. Bioinformatics.

[B101] Gupta N, Mangal N, Biswas S (2005). Evolution and similarity evaluation of protein structures in contact map space. Proteins.

[B102] Zhang GZ, Huang DS (2004). Inter-residue spatial distance map prediction by using integrating GA with RBFNN. Protein Pept Lett.

[B103] Lavine BK, Davidson CE, Breneman C, Kaat W (2004). Genetic algorithms for classification of olfactory stimulants. Methods Mol Biol.

[B104] Goodacre R (2005). Making sense of the metabolome using evolutionary computation: seeing the wood with the trees. J Exp Bot.

[B105] Braun TD, Siegel HJ, Beck N (2001). 6A Comparison of Eleven Static Heuristics for Mapping a Class of Independent Tasks onto Heterogeneous Distributed Computing Systems. J Parall Distrib Comp.

[B106] Press WH, Flannery BP, Teukolsky SA, Vetterling WT (1992). Numerical recipes in C: the art of scientific computing.

